# Zoledronic Acid in the Treatment of Primary Knee Osteoarthritis: A Randomized Clinical Trial

**DOI:** 10.7759/cureus.85087

**Published:** 2025-05-30

**Authors:** Toufiqe Ealahi, Mohammad Abul Kalam Azad, Md. Nazrul Islam, M. Masudul Hassan, Nira Ferdous, Farhana Binty Rashid, Abdul Tazbir

**Affiliations:** 1 Rheumatology, Barishal Medical College Hospital, Barishal, BGD; 2 Rheumatology, Bangladesh Medical University, Dhaka, BGD; 3 Rheumatology, Bangabandhu Medical University, Dhaka, BGD; 4 Internal Medicine, MH Shamorita Hospital and Medical College, Dhaka, BGD; 5 Obstetrics and Gynecology, Dhaka Medical College, Dhaka, BGD; 6 Rheumatology, Enam Medical College, Dhaka, BGD

**Keywords:** bisphosphonate, osteoporosis trial, primary knee osteoarthritis, randomized, randomized trial, zoledronic acid

## Abstract

Background

Pain in primary knee osteoarthritis (OA) is the most disabling symptom. Osteoclast-initiated subchondral bone resorption, marrow edema, and synovitis collectively contribute to pain. Reduction of osteoclast activity by bisphosphonate may be effective in reducing pain.

Objective

This study aims to assess the role of zoledronic acid (ZA) in reducing the pain of primary knee OA.

Methodology

This double-blind, parallel, placebo-controlled randomized clinical trial was conducted in the Bangabandhu Sheikh Mujib Medical University from January 2023 to December 2023. A total of 79 patients were randomly enrolled in the ZA group (*n *= 39) and placebo group (*n *= 40) after having given informed written consent. Each patient was infused with a single infusion of ZA or placebo. Baseline status was measured by three domains (pain, stiffness, and functional status) of the Bangla version of the Western Ontario and McMaster Universities Osteoarthritis Index scale (WOMAC), visual analog scale (VAS), and quality of life by the European Quality of Life-5 Dimensions-5 Levels (EQ-5D-5L). One patient in the ZA group refused to take an infusion.

Patients were followed up at the third and sixth months. During this period, they were allowed to take non-steroidal anti-inflammatory drugs (NSAIDs) on a requirement basis with documentation and advised to undergo non-pharmacological treatment like maintaining ideal body weight, joint protection, and exercise. At the end of the third and sixth months, outcome status was assessed by three domains (pain, stiffness, and functional status) of WOMAC, VAS, and quality of life by EQ-5D-5L. Four patients from the ZA group and one from the placebo group failed to complete follow-up. Finally, the outcome values of 74 patients were compared within and between groups at the end of the third and sixth months. A paired *t*-test and an independent sample *t*-test were applied to compare within and between groups. A *P*-value ≤ 0.05 was considered significant. The study procedure was explained to the patients. Privacy and confidentiality of subjects were maintained.

Results

The study's population was diverse, with an age range of 50-80 years. Sixty (72.5%) were women, and the most common occupations were homemakers (30, 77%) and retired individuals (5, 13%). Vitamin D deficiency was found in 28 (35.4%) subjects, and 30 (38%) had osteoporosis. Within both groups, there was a significant improvement after three months (WOMAC total, *P *= 0.000, and VAS, *P *= 0.001) and six months (WOMAC total, *P *= 0.000, and VAS, *P *= 0.001) and in comparison, of changes between groups, changes in the ZA group were significantly higher (WOMAC total, *P *= 0.000, and VAS, *P *= 0.000) after three and six months. NSAIDs use was considerably lower in the ZA group after three months (*P *= 0.018) and six months (*P *= 0.000). Flu-like symptoms were noted in 14 (37%) patients of the ZA group, and injection site reactions occurred in 5 (12.5%) patients of the placebo group and 2 (5.7 %) in the ZA group.

Conclusions

ZA reduces knee pain, stiffness, and NSAID use, and improves functional status and quality of life at three and six months after a single infusion. With its sustained efficacy and reduced NSAID requirements, this treatment offers a promising alternative for patients and inspires clinicians to explore new avenues in OA management.

## Introduction

Osteoarthritis (OA) is the most common form of arthritis globally. Its prevalence is increasing with age. The global prevalence of knee OA was 22.9% in individuals aged 40 and 16.0% in individuals aged 15, as reported by Cui et al. [1]. In OA knee, the management goal is to focus on symptom and functional improvement. To reduce pain, among other modalities, nonsteroidal anti-inflammatory drugs (NSAIDs) are still first-line agents according to da Costa et al. [2]. However, their use is limited due to the high incidence of gastrointestinal, renal, and cardiovascular side effects [3]. Studies have shown that there was an increased risk of dropouts in trials of NSAIDs and opioids. Chronic use of opioids is associated with an increased risk of fractures, cardiovascular events, dependency, and mortality due to overdose [4]. According to studies, intra-articular steroid injection (IASI) provides short-term relief for one to eight weeks, and the usual practice is limited to four injections annually [5]. A radiological study showed that documented adverse joint events after IASI include accelerated OA progression, osteonecrosis, and rapid joint destruction with bone loss [6]. So, no recommended pharmacological treatment provides sustained symptomatic relief and fewer treatment complications. A guideline-based treatment of multi-morbid and elderly patients represents a particular challenge. In many studies on which the guidelines were based, detailed evaluation of the multi-morbid and elderly patient is often avoided to make the study design less complicated [7]. Authors have reported that bisphosphonates can reduce pain in many experimental drugs, while others have suggested that bisphosphonates can ameliorate the structural findings observed in patients with OA [8]. Both oral (alendronate sodium) and parenteral (zoledronic acid (ZA)) have clinical efficacy in reducing symptoms and structural progression [9]. We had preferred ZA for better compliance in our study. OA is characterized by focal cartilage loss, osteophyte formation, subchondral bony changes, and, less commonly, synovitis with involvement of periarticular structures. In subchondral bone, there is a decreased number and thinning of tibial cancellous trabeculae and localized subchondral osteoporosis (OP) in knee OA [10]. Several mechanisms, including abnormal osteoclast-mediated bone resorption, are responsible for OA's disease progression and pain [11]. It is also mentioned in the study by Zhu et al. [12] that the osteoclasts secrete netrin-1 to induce sensory nerve axonal growth in the subchondral bone, aggravating pain. Therefore, regulating osteoclast-mediated inflammatory pathways and modifying bone metabolism with bisphosphonates may help treat OA [13]. At the age of 50 and above, another prevalent disease is OP, both in the general population and patients with OA [14]. In this case, bisphosphonate may be an effective single option in treating patients with OA with OP [15]. The primary aim of this study was to evaluate the efficacy of ZA in treating primary knee OA. The secondary objectives were to assess the quality of life in primary knee OA, observe the prevalence rate of OP in OA patients, and observe the vitamin D status in OA patients. There are several pharmacological modalities of treatment for knee OA, like frequent NSAIDs, opioids, and intra-articular steroids, which can patients to compliance issues. There is a need for compliance-friendly medication for this condition. Therefore, a single shot of ZA, whose efficacy lasts for a year, may be an option for the treatment of knee OA. Several studies reported mixed outcomes. This randomized controlled trial aimed to evaluate the efficacy of ZA in treating knee OA.

## Materials and methods

Study design and setting

This experimental study (randomized controlled trial) was conducted in the Outpatient Department of Rheumatology, Bangabandhu Sheikh Mujib Medical University (BSMMU), from July 2022 to December 2023. Ethical clearance was obtained from the Institutional Review Board (IRB) of BSMMU, approval number BSMMU/2023/930, and was also registered at ClinicalTrials.gov (NCT06051344).

Inclusion criteria

Patients who had knee pain and attended rheumatology outdoors were included. The study was conducted using convenience sampling, with a sample size of 33. The sample size calculation formula was, *Z*α = 1.96, *Z* distribution at 95% confidence level, *Z*(1 - β) =1.28 (at 90% power), standard deviation (SD) = 25 mm, and *d* (effect size) = 20 mm on VAS as pain intensity (from a previous study done by Laslett et al. [8]), *n* = 33, considering the 10% dropout adjusted sample size, *N* = *n*/(1 - *d*) (dropout) = 33/0.9 = 37, for each group. Patients aged ≥ 50 years with knee OA diagnosed according to the American College of Rheumatology (ACR) criteria, who consented to participate in the study, were included.

Exclusion criteria

Patients with known inflammatory arthritis, a body mass index (BMI) ≥ 40 kg/m², prior diagnosis of cancer, prior use of bisphosphonates, use of intra-articular corticosteroid or hyaluronic acid preparations within the past three months, metabolic causes of OA (acromegaly, hypothyroidism, diabetes mellitus), mechanical causes of OA (hypermobility syndromes, avascular necrosis, congenital dislocations, limb-length inequality), history of knee joint fracture or surgery, Charcot joint, serum calcium >11.0 mg/dL or <8.0 mg/dL, creatinine clearance <35 mL/min, poor dental hygiene or planned invasive dental procedures, and Kellgren and Lawrence grade 4 were excluded.

Study procedure

**Figure 1:**
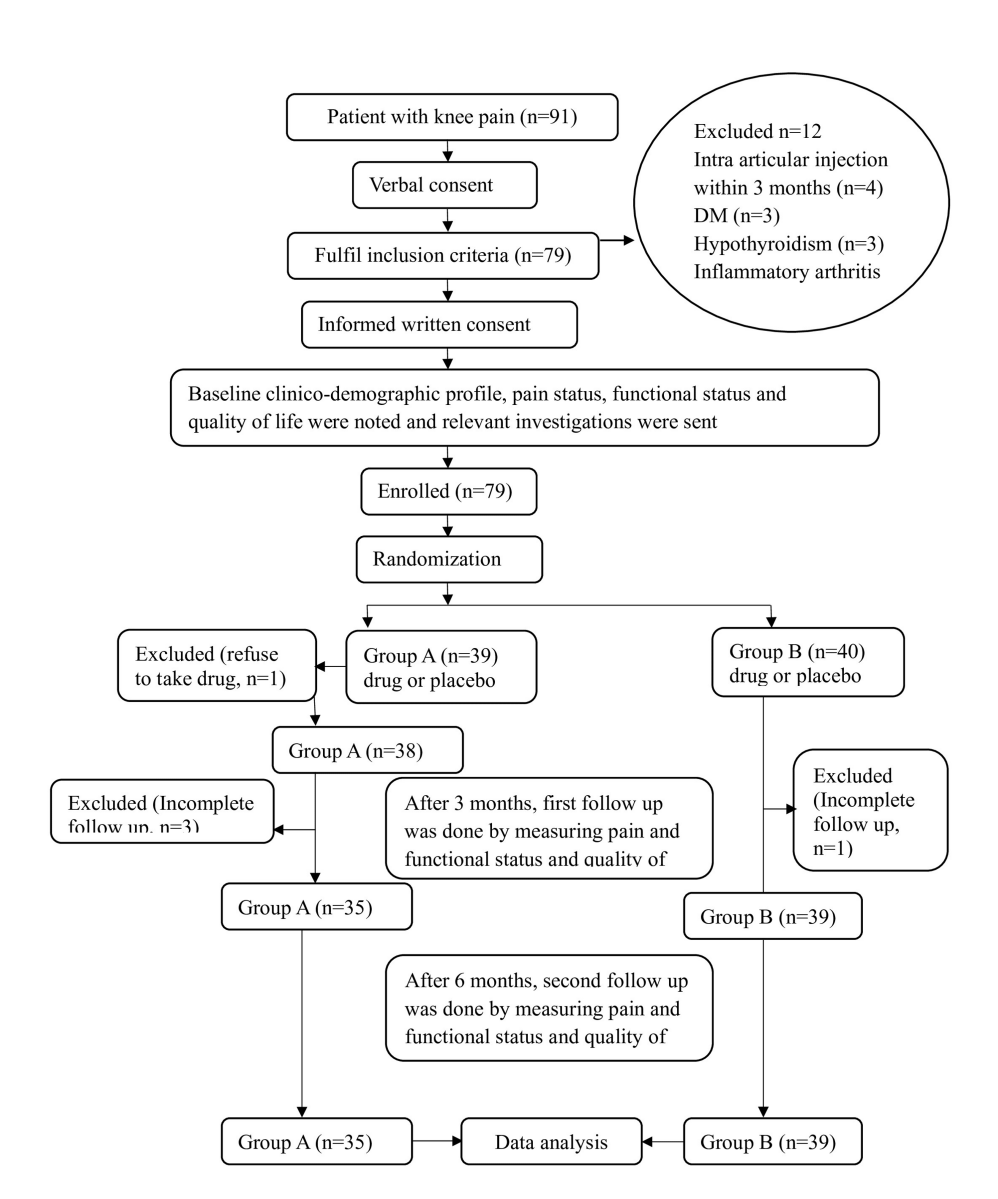
Flowchart of study subjects. Image credit: Toufiqe Ealahi.

This randomized controlled trial was conducted at the rheumatology outpatient department. At first, verbal consent was taken from the patient with knee pain. Ninety-one patients had given verbal consent and were informed about the aims and benefits of the study. ACR clinical and radiological criteria [16] diagnosed the primary knee OA; subsequently, history, physical examination, and investigations were done to evaluate exclusion criteria. Seventy-nine patients were eligible, and four patients were excluded due to intra-articular injection within three months, 3 for DM, 3 for hypothyroidism, and 2 for inflammatory arthritis. Eligible patients were provided with an informed written consent form. Baseline pain status was measured by the Bangla version of the WOMAC scale [17], VAS, and quality of life by the European Quality of Life-5 Dimensions-5 Levels (EQ-5D-5L) [18]. The flowchart of study subjects and their follow-up is shown in Figure 1.

Intervention and follow-up

Randomization was performed using a randomization table generated via the website http://ctrandomization.cancer.gov/tool. Participants were assigned to two groups (Group A and Group B), with each group receiving either a single infusion of ZA or a placebo. One patient in Group A declined to receive the injection.

Group A received a single infusion of QKHO73, a coded drug, and Group B received PTZI01, a coded drug. Five patients dropped out: four were lost to follow-up, and one refused to take the infusion. The code was broken; QKHO73 was ZA, manufactured by Opsonin Pharma Ltd., and PTZI01 was the placebo (normal saline) with the same color and size bottle, manufactured by Opsonin Pharma Ltd., Bangladesh. Analysis was done on 74 participants.

Patients were followed up at the third and sixth months. During this period, they were allowed to take NSAIDs on a requirement basis with documentation and advised to undergo non-pharmacological treatment like maintaining ideal body weight, joint protection, and exercise. At the end of the third and sixth months, pain status was reassessed using the WOMAC and VAS scales, and quality of life was evaluated using the EQ-5D-5L.

After three months, three patients from Group A and one from Group B missed their first follow-up and were excluded from the study.

Research Instruments

Pain was assessed using WOMAC and VAS; functional status was assessed using WOMAC; and quality of life was assessed using EQ-5D. A flowchart showing the screening of the participants is presented in Figure 1.

Statistical analysis

Data were analyzed using SPSS, version 25 (IBM Corp., Armonk, NY). Qualitative data were expressed in percentages. Quantitative data were expressed as mean ± SD. The chi-square test and Fisher’s exact test were carried out for qualitative variables. Independent sample t-tests were done to see the changes in outcome variables within groups and between groups at the end of the third month and sixth month. *P* ≤ 0.05 was considered significant.

Quality control strategy

A pilot study was conducted on ten patients before the actual enrollment. Relevant modifications to the datasheet were made based on the pilot results. An evaluation was conducted after collecting data from 25% of the patients to ensure consistency in the study. The guide and co-guides regularly reviewed the study status and data. The placebo or drug was infused under the direct supervision of the investigator. It was ensured that the patient did not take any other medication suggestions from the investigator. Painkillers that were taken by patients should be properly documented by themselves and also informed by the investigator. To prevent loss to follow-up, the telephone numbers of both the patient and caregiver, as well as their present and permanent addresses, were meticulously recorded. Ten percent of the data was randomly checked by the guide. After collection, all data were reviewed for inadequacy, irrelevance, and inconsistency. Irrelevant and inconsistent data were discarded.

Ethical implications

The aims and objectives of the study, along with its procedure, risks, and benefits, were explained to the patients, and informed written consent was obtained from each patient before enrollment. Privacy and confidentiality were strictly maintained. Only the investigator and the guide had access to the data, except for law-enforcing persons in special circumstances. Data might be exposed abroad for academic purposes only while maintaining anonymity. Every patient had the right to withdraw from the study at any time without losing access to optimal medical care. This study was conducted free from any economic benefits or influences.

## Results

**Figure 2:**
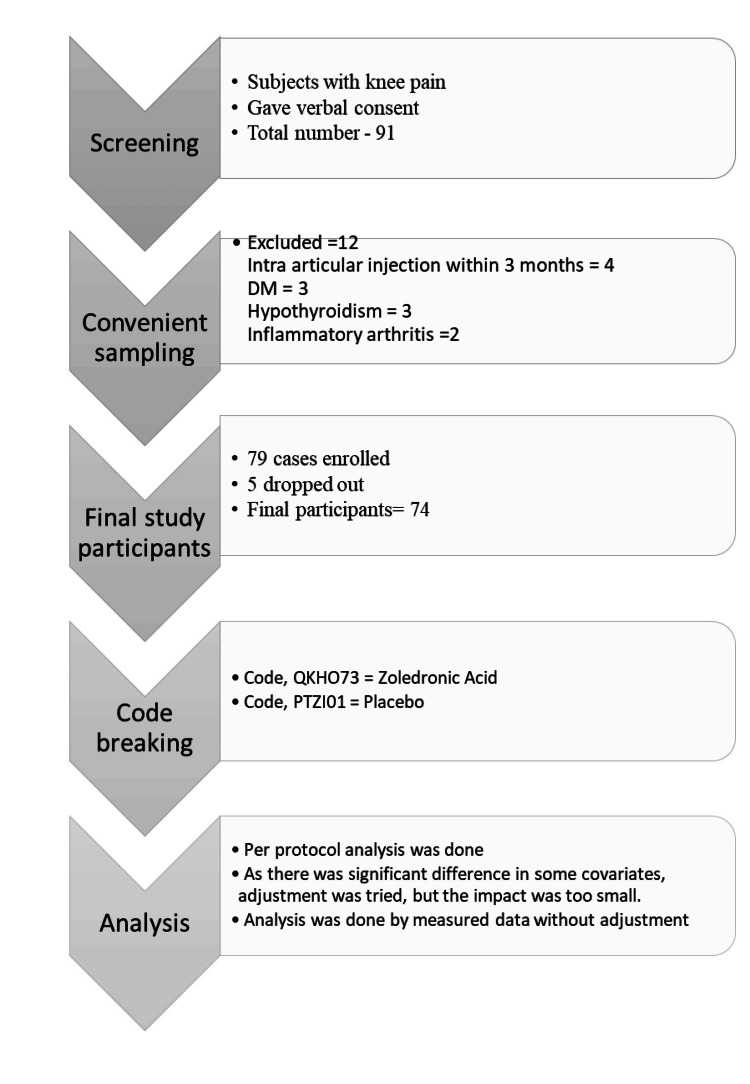
A flowchart showing the screening of the participants. Image credit: Toufiqe Ealahi.

Ninety-one patients aged ≥50 and of any gender suffering from knee pain were screened for primary knee OA. Twelve were excluded: four had intra-articular injections within three months, three had DM, three had hypothyroidism, and two had inflammatory arthritis. The remaining 79 patients were enrolled and randomized into two groups (Group A and Group B). A flowchart of the study subjects is shown in Figure 2.

Group A received a single infusion of QKHO73, a coded drug, and Group B received PTZI01, a coded drug. Five patients dropped out: four were lost to follow-up, and one refused to take the infusion. The code was broken; QKHO73 was ZA, and PTZI01 was the placebo. Analysis was done on 74 participants.

Demographic, clinical characteristics, and laboratory parameters of the study subjects (n = 79). Among the 79 participants, the age range was 50-80 years. Sixty (72.5%) were women. Nineteen (49%) had primary education. The prevalent occupations were housewife, 30 (77%), and retired, 5 (13%). Six (7.6%) were smokers, and 24 (30.3%) were current users of other forms of tobacco. Among the females, 22 (36.67%) had a history of hormonal contraceptive use, and the average number of children was around four. The mean disease duration of OA was 37.16 ± 40.67 months. The predominantly involved knees assessed for outcomes were right knees in 40 (50.6%) and left knees in 39 (49.4%) patients. Thirty-four (43%) patients were hypertensive; 40 (50.6%) were obese; 15 (19%) were overweight; 17 (21.5%) normal weight; and 7 (8.9%) were underweight. Vitamin D deficiency was found in 28 (35.4%) subjects, and 30 (38%) had OP. Baseline clinicodemographic and laboratory findings are shown in Tables 1-2, respectively.

**Table 1:** Baseline clinicodemographic characteristics (n = 79). n, number; BMI, body mass index; HRT, hormone replacement therapy; SD, standard deviation; ZA, zoledronic acid *The test is significant. **t-value. ***X^2^ value, significant.

Patient characteristics	ZA group, *n* = 39 (%)	Placebo group, *n* = 40 (%)	t-/*X*^2-^value	*P*-value
Age (mean ± SD) (years)	61.6 ± 7.6	57 ± 8.3	1.820**	0.020
50-65 years	27 (69.2)	36 (90.0)	5.270**	0.468
66-80 years	12 (30.8)	4 (10.0)		
M/F	8 (20.5)/31 (79.5)	11 (27.5)/29 (72.5)	0.538**	0.468
BMI (mean ± SD) (kg/m²)	23.6 ± 4.2	26.8 ± 4.4	6.950**	0.001
Education Illiterate	12 (30.8)	4 (10.0)	6.210***	0.025
Primary	19 (48.7)	21 (52.5)		
Secondary or more	8 (20.5)	15 (37.5)		
Occupation housewife	30 (76.9)	27 (67.5)	1.290**	0.26
Retired	5 (12.8)	9 (22.5)		
Others	4 ((10.3)	4 (10.0)		
Smoking	4 (10.3)	2 (5.0)	0.780**	0.370
Tobacco in another form	18 (46.2)	6 (15.0)	9.090**	0.002*
History of HRT in females	7 (22.6)	15 (51.7)	3.750**	0.053
Child numbers in females (mean ± SD)	4.8 ± 1.4	3.5 ± 1.2	4.440**	0.004
Target joint: Right/left	21 (53.8)/18 (46.2)	19 (47.5)/21 (52.5)	0.320***	0.573
Duration (mean ± SD) (months)	33.4 ± 38.0	40.8 ± 43.3	0.800**	0.425

**Table 2:** Baseline laboratory findings of the study subjects (n = 79). *, independent sample t-test; n, number; SD, standard deviation; WOMAC, Western Ontario and McMaster Universities Osteoarthritis Index; EQ-5D-5L, European Quality of Life-5 Dimensions-5 Levels; LSS, level sum score; VAS, visual analog scale; ZA, zoledronic acid; ALT, alanine transaminase; ESR, erythrocyte sedimentation rate; **, *t*-value; ***, *X*2 value; BMD, bone mineral density

Parameters	ZA group (*n* = 39) (%)	Placebo group (*n* = 40) (%)	*t*-**/*X*^2^-value***	*P*-value*
X-ray (KL grading)				
Grade 0	3 (8.6)	5 (12.8)	0.370***	0.5439
Grade 1	23 (66.7)	25 (64.1)	-	-
Grade 2	4 (11.4)	4 (10.3)	-	-
Grade 3	5 (14.3)	5 (12.8)	-	-
Vitamin D				
Deficient	14 (35.9)	14 (35.9)	1.806***	0.873
Insufficient	16 (41.0)	21 (52.5)	-	-
Sufficient	9 (23.1)	5 (12.5)	-	-
BMD (mean ± SD) (Gm/cm²)				
Lumber vertebra	0.88 ± 0.1	0.82 ± 0.2	1.670**	0.000*
Right femoral neck	0.94 ± 0.1	0.88 ± 0.2	3.360**	0.000*
Left femoral neck	0.93 ± 0.1	0.88 ± 0.1	2.220**	0.000*
BMD T-score				
Osteoporosis (≤-2.5)	22 (62.9)	8 (20.5)	13.930**	0.0003*
Osteopenia (-2.4 to 1)	9 (25.7)	19 (48.7)	-	-
Normal (>1)	4 (11.4)	12 (30.8)	-	-
Hb (mean ± SD) (g/dL)	11.5 ± 1.4	12.0 ± 1.5	2.240**	0.089
ESR (mean ± SD) mm in the first hour	29.7 ± 13.3	26.3 ± 16.7	0.880**	0.305
ALT (mean ± SD) (U/L)	27.67 ± 8.4	26.67 ± 9.3	0.000	0.608
Serum creatinine (mean ± SD) (mg/dL)	0.8 ± 0.2	0.9 ± 0.2	2.220	0.566

At enrollment, the WOMAC pain score in the ZA vs. placebo group was 27.0 ± 4.3 vs. 23.3 ± 5.2, WOMAC stiffness score 9.6 ± 2.6 vs. 8.7 ± 2.3, WOMAC functional status score 99.5 ± 13.4 vs. 88.1 ± 17.4, and WOMAC total score 136.1 ± 19.4 vs. 120 ± 23.1. The pain score on the VAS in the ZA vs. placebo group was 64.1 ± 11.6 vs. 57.3 ± 12.4. In the EQ-5D-5L, EQ-VAS, and baseline level sum scores (LSSs) were 36.6 ± 11.4 vs. 43.1 ± 12.3, 11.6 ± 2.3 vs. 11.6 ± 2.3, and 10.8 ± 2.2 in the ZA vs. placebo group, respectively. Baseline values of WOMAC pain, stiffness, functional status, WOMAC total, VAS, EQ-VAS, and LSS in EQ-5D-5L are shown in Table 3.

**Table 3:** Comparison of baseline values of WOMAC pain, stiffness, functional status, and WOMAC total, VAS, EQ-VAS, and LSS in EQ-5D-5L and quality-of-life scores of two groups (n = 79). *, independent sample t-test; n, number; SD, standard deviation; WOMAC, Western Ontario and McMaster Universities Osteoarthritis Index; EQ-5D-5L, European Quality of Life-5 Dimensions-5 Levels; LSS, level sum score; VAS, visual analog scale; ZA, zoledronic acid *The test is significant. **t-value.

Parameter	ZA group (*n* = 39) (mean ± SD)	Placebo group (*n* = 40) (mean ± SD)	*t*-value	*P*-value*
WOMAC				
Pain total (0-50)	27.0 ± 4.3	23.3 ± 5.2	3.44**	0.001*
Stiffness total (0-20)	9.6 ± 2.6	8.7 ± 2.3	1.63**	0.086*
Functional status total (0-170)	99.5 ± 13.4	88.1 ± 17.4	3.25**	0.002*
WOMAC total (0-240)	136.1 ± 19.4	120 ± 23.1	3.35**	0.001*
VAS (0-100)	64.1 ± 11.6	57.3 ± 12.4	2.52**	0.013*
EQ-VAS (0-100)	36.6 ± 11.4	43.1 ± 12.3	2.43**	0.013*
EQ-5D-5L (5-25)	11.6 ± 2.3	10.8 ± 2.2	1.58**	0.138

Comparison of mean changes in WOMAC pain, stiffness, functional status, WOMAC total, VAS, EQ-VAS, and LSS in EQ-5D-5L between the ZA and placebo groups at three and four to six months, which are statistically significant, is shown in Table 4.

**Table 4:** Comparison of changes in WOMAC pain, stiffness, functional status, and WOMAC total, VAS, EQ-VAS, and LSS in EQ-5D-5L between groups at three months and six months (4-6) (n = 74). **, independent sample t-test; n, number; SD, standard deviation; WOMAC, Western Ontario and McMaster Universities Osteoarthritis Index; EQ-5D-5L, European Quality of Life-5 Dimensions-5 Levels; LSS, level sum score; VAS, visual analog scale; ZA, zoledronic acid *The test is significant.

Follow-up	At three months			Six months		
Parameter	ZA (*n* = 35)	Placebo group (*n* = 39)	*P*-value**	ZA (*n* = 35)	Placebo (*n* = 39)	*P*-value**
WOMAC						
Pain total (0-50)	-0.0 ± 1.7	-4.6 ± 1.4	0.000*	-4.9 ± 1.5	-1.9 ± 1.6	0.000*
Stiffness total (0-20)	-0.9 ± 0.9	-1.4 ± 1.0	0.019*	-0.6 ± 1.1	-0.1 ± 0.4	0.012*
Functional status total (0-170)	-9.5 ± 7.2	-15.6 ± 4.4	0.006*	-16.3 ± 4.7	-9.1 ± 3.2	0.006*
WOMAC total (0-240)	-29.0 ± 7.3	-21.5 ± 5.6	0.000*	-22.1 ± 6.2	-11.1 ± 4.4	0.000*
VAS (0-100)	-4.6 ± 5.1	-9.7 ± 1.6	0.000*	-11.4 ± 3.6	-6.9 ± 4.7	0.000*
EQ-VAS (0-100)	14.6 ± 5.1	-9.5 ± 3.9	0.000*	10.9 ± 0.7	6.7 ± 4.7	0.000*

The total amount of NSAIDs (naproxen potassium) used by patients up to three months was 26.9 ± 6.7 g vs. 31.3 ± 8.5 g in the ZA vs. placebo group. From four to six months, usage was 10.2 ± 2.8 g vs. 25.6 ± 7.1 g in the ZA vs. placebo group, as shown in Table 5.

**Table 5:** The total amount of NSAIDs at 0-3 and 4-6 months in between groups (n = 74). *, independent sample t-test; NSAIDs, non-steroidal anti-inflammatory drug; n, number; SD, standard deviation; ZA, zoledronic acid **The test is significant.

Amount of naproxen potassium	ZA group (*n* = 35)	Placebo group (*n* = 39)	*t*-value	*P*-value*
In 0-3 months (mean ± SD) (g)	26.9 ± 6.7	31.3 ± 8.5	2.46	0.018
At 4-6 months (mean ± SD) (g)	10.2 ± 2.8	25.6 ± 7.1	12.03	0.000**

In this study, flu-like symptoms occurred in 14 patients in the ZA group. Injection site reactions occurred in 5 patients in the placebo group and 2 in the ZA group, as shown in Table 6.

**Table 6:** Adverse events in both the ZA and placebo groups. n, number; ZA, zoledronic acid

Parameter	ZA group (*n *= 38)	Placebo group (*n* = 40)
Flu-like symptoms, *n* (%)	14 (38.6)	0 (00)
Infusion site reaction, *n* (%)	2 (5.7)	5 (12.5)

## Discussion

Pain is the most disabling symptom of knee OA. Several factors play a role in pain in knee OA, including subchondral bone resorption. This randomized, placebo-controlled study was hypothesized to determine the efficacy of ZA (an anti-resorptive drug) in the pain outcome of primary knee OA.

Most patients in both groups were female, similar to a previous study in Bangladesh [19], and another study showed the same result [20]. OA is more prevalent in females due to thinner cartilage, the tendency to varus malalignment, and the steep decline of sex hormone levels at menopause [21]. The mean age of the treatment group was 61 ± 7.6 years, and in the placebo group, it was a little lower, 57.4 ± 8.2 years, similar to a previous Australian study [8]. Most patients, around 91%, are distributed between 50 and 70 years, and have a reduced frequency above this age. A similar observation was seen in a previous study from the Global Burden of Disease Study 2019 [22]. Most of the patients were obese (50.6%) according to Asian BMI criteria, and housewife was the most common occupation in both groups of our study, consistent with findings from other Asian studies [19].

A profession related to kneeling, squatting, lifting, and climbing stairs and ladders is highly associated with knee OA [23]. Around 50% of subjects had primary education, and 20% were illiterate. Lower educational status is associated with a higher prevalence of knee OA and symptoms [24-25]. Only six people in our study were smokers. There is a negative relation between smoking and OA, as nicotine has some chondroprotective effects [26]. However, 24 subjects in our study had a history of taking smokeless tobacco like Jorda. There is no study regarding the impact of smokeless tobacco on OA. Seventeen patients (21.5%) had a bilateral presentation, with the most symptomatic knee on the right. The left was almost similar (50.6% and 49.4%), matching a previous study [27]. Vitamin D deficiency was found in 28 (35.4%) patients, which was higher than an earlier Indian study (24.49%) [14]. OP was found in 30 (38%) patients, which was almost similar (32.7%) in a previous study [14].

We have used the Bangla version of WOMAC to assess pain, stiffness, and functional status, VAS for pain, and EQ-5D-5L to determine quality of life. In both groups, there was a significant improvement after three months and six months in all sections of WOMAC, like pain, stiffness, functional status, and total WOMAC scores, VAS, EQ-5D-5L, and EQ VAS, but higher in the ZA group. A similar observation was made in a study by Cai et al. using Zolindronate [28]. They found an identical reduction in signs and symptoms after using the WOMAC scale in both treatment and placebo groups. They hypothesized that the expectation bias of the patient over the placebo might be the cause. Different observations were found in the previous ZA trials; only a significant reduction was found in the pain scale at six months in the ZA group. No significant improvements were found in any group at three months [8]. In our study, though there were significant improvements in WOMAC pain, stiffness, and function, VAS within groups at three and six months. A previous study found a similar observation [28]. However, we have found significant differences in the mean scores between groups at three and six months; changes in scores were significantly higher in the ZA group at both time points. NSAID use was significantly lower in the ZA group, which was different from the previous Iranian study and might be a cause of improvement in the placebo group [29]. In this study, flu-like symptoms were observed in 11 patients in the ZA group, which was lower than reported in previous studies [8,28].

Strengths

This was a double-blinded, randomized, prospective study. We used the WOMAC 3.1 Bangla version and instructed the patients to use a single variety of NSAIDs on a requirement basis. We performed two follow-ups at the third and sixth months. To the best of our knowledge, this was the first single-center study in Bangladesh evaluating the efficacy of ZA in primary knee OA.

Limitations

We were unable to isolate the drug effect from the placebo effect and other dietary influences. Additionally, we could not extend the follow-up to 12 months to assess the persistence of efficacy.

## Conclusions

A significant reduction in pain and total NSAID use, along with improvement in joint function and quality of life, was observed at the third and sixth months following a single infusion of ZA. We recommend further studies with longer follow-up periods to evaluate the persistence of its effects.
